# Rapid cross-border emergence of NDM-5-producing *Escherichia coli* in the European Union/European Economic Area, 2012 to June 2022

**DOI:** 10.2807/1560-7917.ES.2023.28.19.2300209

**Published:** 2023-05-11

**Authors:** Marius Linkevicius, Rémy A Bonnin, Erik Alm, Olov Svartström, Petra Apfalter, Rainer Hartl, Henrik Hasman, Louise Roer, Kati Räisänen, Laurent Dortet, Niels Pfennigwerth, Jörg B Hans, Ákos Tóth, Lilla Buzgó, Martin Cormican, Niall Delappe, Monica Monaco, Maria Giufrè, Antoni PA Hendrickx, Ørjan Samuelsen, Anna K Pöntinen, Manuela Caniça, Vera Manageiro, Jesús Oteo-Iglesias, María Pérez-Vázquez, Karin Westmo, Barbro Mäkitalo, Daniel Palm, Dominique L Monnet, Anke Kohlenberg

**Affiliations:** 1 European Centre for Disease Prevention and Control, Stockholm, Sweden; 2 French National Reference Center for Antimicrobial Resistance, INSERM UMR 1184, Paris-Saclay University, Bicêtre Hospital, Assistance Publique des Hôpitaux de Paris, Paris, France; 3 Austrian National Reference Centre for Antimicrobial Resistance, Ordensklinikum Linz Elisabethinen, Linz, Austria; 4 Department of Bacteria, Parasites and Fungi, Statens Serum Institut, Copenhagen, Denmark; 5 Department of Health Security, Finnish Institute for Health and Welfare, Helsinki, Finland; 6 National Reference Centre for multidrug-resistant Gram-negative bacteria, Ruhr University Bochum, Bochum, Germany; 7 National Public Health Centre, Budapest, Hungary; 8 University of Galway, Galway, Ireland; 9 Department of Infectious Diseases, Istituto Superiore di Sanità, Rome, Italy; 10 Centre for Infectious Disease Control (CIb), National Institute for Public Health and the Environment, Bilthoven, the Netherlands; 11 Norwegian National Advisory Unit on Detection of Antimicrobial Resistance, University Hospital of North Norway, Tromsø, Norway; 12 Department of Pharmacy, Faculty of Health Sciences, UiT The Arctic University of Norway, Tromsø, Norway; 13 Department of Biostatistics, Faculty of Medicine, University of Oslo, Oslo, Norway; 14 National Reference Laboratory of Antibiotic Resistances and Healthcare Associated Infections, Department of Infectious Diseases, National Institute of Health Dr. Ricardo Jorge, Lisbon, Portugal; 15 Laboratorio de Referencia e Investigación en Resistencia a Antibióticos del Centro Nacional de Microbiología and CIBERINFEC, Instituto de Salud Carlos III, Madrid, Spain; 16 Public Health Agency of Sweden, Stockholm, Sweden

**Keywords:** Carbapenem-resistant Enterobacterales, carbapenemase, NDM-5, *Escherichia coli*, surveillance, whole genome sequencing, cross-border import

## Abstract

Whole genome sequencing data of 874 *Escherichia coli* isolates carrying *bla*_NDM-5_ from 13 European Union/European Economic Area countries between 2012 and June 2022 showed the predominance of sequence types ST167, ST405, ST410, ST361 and ST648, and an increasing frequency of detection. Nearly a third (30.6%) of these isolates were associated with infections and more than half (58.2%) were predicted to be multidrug-resistant. Further spread of *E. coli* carrying *bla*_NDM-5_ would leave limited treatment options for serious *E. coli* infections.

Preliminary unpublished results of a survey of carbapenem- and/or colistin-resistant Enterobacterales (CCRE survey) [1] in 36 European countries showed that New Delhi metallo-β-lactamase (NDM)-5 was the most frequently reported carbapenemase in *Escherichia coli* with detection of *bla*_NDM-5_ in 62 (30.8%) of 201 carbapenemase-producing *E. coli* isolates collected in 2019. These 62 *E. coli* isolates were detected in 15 countries and involved various *E. coli* sequence types (STs), some of which are described as high-risk STs for extraintestinal infections [2]. These findings were of concern and warranted further investigation with the aim to confirm the extent of spread and describe the epidemiological and microbiological characteristics of the detected isolates.

## Data collection and analysis

The European Centre for Disease Prevention and Control requested, via its EpiPulse platform, whole genome sequencing (WGS) and epidemiological data on *E. coli* carrying *bla*_NDM-5_ from European Union (EU)/European Economic Area (EEA) countries. In reply, data for 905 *E. coli* isolates were received from 13 countries. Twenty-nine isolates did not fulfil the quality criteria (assembled genome 3.7–6.4 Mbp with ≥ 90% core genome loci covered using the EnteroBase core genome multilocus sequence typing scheme (cgMLST [3]), and two isolates did not carry *bla*_NDM-5_. Therefore, data from 874 isolates from the period 2012 to June 2022 were included in the analysis (Table); these isolates are hereafter referred to as being from ‘national collections’. The data for this study were deposited in the European Nucleotide Archive under accession numbers PRJEB27363, PRJEB36710, PRJEB45009, PRJEB56146, PRJEB61153 and PRJNA845120.

**Table:** Dominant sequence types of *Escherichia coli* isolates carrying *bla*_NDM-5_ submitted from national collections, by country and period covered, EU/EEA, 2012–June 2022 (n = 874)

*E. coli* sequence type	Number of isolates by country (period covered)													
	AT (2019–2022)	DE (2019)	DK (2015–2022)	ES (2014–2021)	FI (2016–2022)	FR (2017–2022)	HU (2020)	IE (2017–2022)	IT (2017–2018)	NL (2012–2022)	NO (2021–2022)	PT (2017–2020)	SE (2021–2022)	Total (2012–2022)
ST167	0	2	23	1	5	95	0	8	4	42	3	6	11	**200**
ST405	0	2	8	2	8	49	0	15	0	24	0	0	7	**115**
ST410	0	2	7	0	3	54	1	8	2	13	0	1	5	**96**
ST361	0	1	9	0	3	42	0	5	0	6	2	0	2	**70**
ST648	0	1	5	0	6	18	0	11	0	12	1	0	11	**65**
Other STs	4	2	30	2	20	145	1	33	0	61	6	6	18	**328**
**Total**	**4**	**10**	**82**	**5**	**45**	**403**	**2**	**80**	**6**	**158**	**12**	**13**	**54**	**874**

AT: Austria; DE: Germany; DK: Denmark; EEA: European Economic Area: ES: Spain; EU: European Union; FI: Finland; FR: France; HU: Hungary; IE: Ireland; IT: Italy; NL: the Netherlands; NO: Norway; PT: Portugal; SE: Sweden ST: sequence type.

We downloaded WGS data from the National Center for Biotechnology Information (NCBI) Pathogen Detection on 20 July 2022 and included an additional 2,561 *E. coli* isolates carrying *bla*_NDM-5_ after quality control using the same criteria as described above. We determined the STs with the MLST 2.0 tool from the Danish Technical University (DTU) (version 2.0.9, database version 26 July 2022, using the Achtman MLST scheme for *E. coli*) [4]. In line with previously published definitions [5], major STs were defined as representing > 10% and minor STs as 5–10% of the isolates from national collections. Resistance genes were determined using *E. coli* analysis plugin of BioNumerics 7.6.3 (Applied Maths NV/bioMérieux, Sint-Martens-Latem, Belgium) with thresholds of ≥ 90% sequence identity and ≥ 60% sequence length for gene coverage. Fluoroquinolone resistance point mutations were detected using the PointFinder tool from DTU (reference sequences collected on 29 September 2022) [6]. Clusters of related isolates were determined with a threshold of 10 allelic differences based on previously published studies [7] and using the EnteroBase cgMLST scheme [3].

The epidemiological variables collected for isolates from national collections included year and month of sample collection, the location of the healthcare institution submitting the sample (city and National Territorial Units for Statistics level 2 region), the sample type (screening or clinical sample) and site of sampling for clinical samples, the clinical relevance (infection or carriage), the status of the patient (inpatient or outpatient), the type of acquisition (community- or healthcare-associated), age and sex of the patient, travel/hospitalisation and country of travel/hospitalisation in the 6 months before sampling and an epidemiological link to another patient.

## Distribution of sequence types

**Figure 1:**
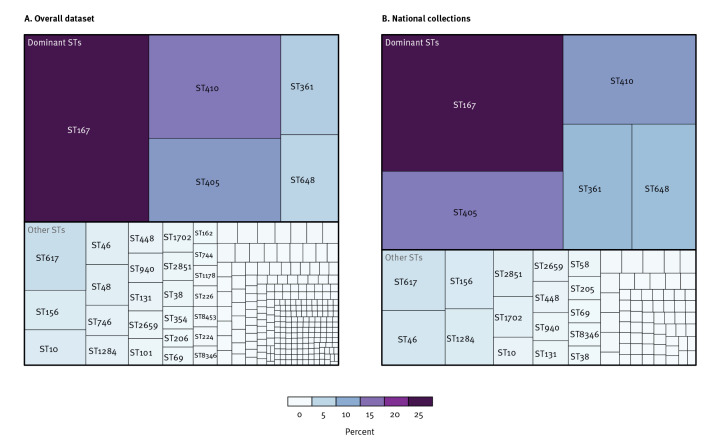
Distribution of sequence types of *Escherichia coli* isolates carrying *bla*_NDM-5_ from two datasets: overall dataset (n = 267)^a^, and national collections (n = 83), EU/EEA, 2012–June 2022

EEA: European Economic Area; EU: European Union; ST: sequence type.

**Figure 2:**
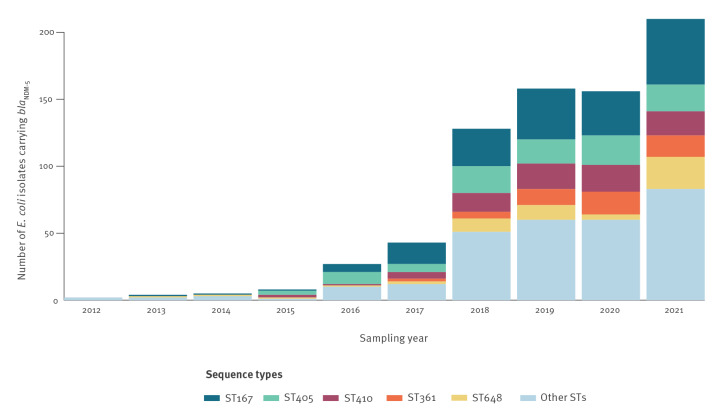
Frequency of sequence types of *Escherichia coli* isolates carrying *bla*_NDM-5_ over time by year of sampling in the dataset from national collections, EU/EEA, 2012–2021^a ^(n = 741)

EEA: European Economic Area; EU: European Union; ST: sequence type.

^a^ The time distribution illustrated above should not be interpreted as an epidemic curve as year-to-year variation is probably affected by detection and reporting biases and may not reflect true temporal trends in incidence. Isolates from 2022 (n = 133) are not displayed because data for that year were incomplete at the time of analysis. Seventy STs other than the dominant ones are grouped as “Other STs”.

The overall dataset included 3,435 *E. coli* isolates carrying *bla*_NDM-5_ belonging to 267 STs. Of these, 83 STs were present in the 874 isolates from national collections, with five predominant STs: ST167 (n = 200; 22.9%), ST405 (n = 115; 13.2%), ST410 (n = 96; 11.0%), ST361 (n = 70; 8.0%) and ST648 (n = 65; 7.4%) (Figure 1). Based on the national collections and the definitions outlined above, ST167, ST405 and ST410 were classified as major STs and ST361 and ST648 as minor STs, and they were grouped together as dominant STs for further analyses. The five dominant STs were detected in all countries that submitted more than 20 isolates, i.e. Denmark, Finland, France, Ireland, the Netherlands and Sweden (Table). The number of detected *E. coli* isolates carrying *bla*_NDM-5_ increased over time (Figure 2). Isolates from national collections (n = 500) were involved in 162 clusters of which 114 were multi-country clusters.

Isolates without a specified ST were excluded (n = 53 for overall dataset and n = 35 for national collections).

^a^ The overall dataset included isolates from the following countries: Argentina, Australia, Austria, Bangladesh, Cambodia, Canada, Chile, China, Czechia, Denmark, Egypt, Finland, France, Germany, Hungary, India, Ireland, Israel, Italy, Japan, Kenya, Laos, Lebanon, Malawi, Myanmar/Burma, Nepal, the Netherlands, Niger, Nigeria, Norway, Pakistan, Portugal, Qatar, Singapore, South Korea, Spain, Sweden, Switzerland, Taiwan, Tanzania, Thailand, United Kingdom, United States and Vietnam, collected or uploaded to NCBI between 2014 and 2022.

## Resistance determinants

**Figure 3:**
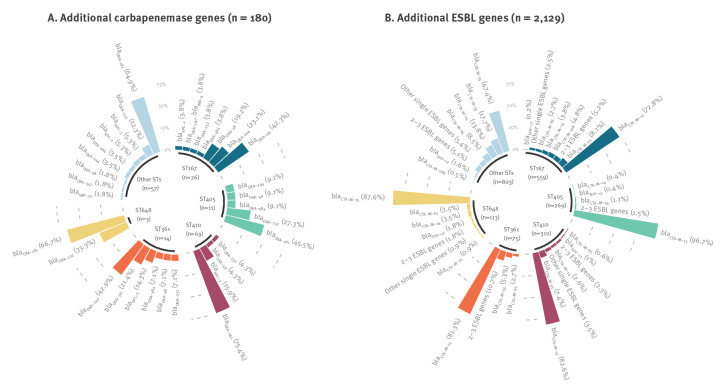
Distribution of *Escherichia coli* isolates with *bla*_NDM-5_ co-carrying additional carbapenemase (n = 180) or ESBL (n = 2,129) genes, by dominant sequence types from the overall dataset^a^

ESBL: extended-spectrum β-lactamase; ST: sequence type.

In the overall dataset, a small percentage (n = 180; 5.2%) of *E. coli* isolates carrying *bla*_NDM-5_ harboured additional carbapenemase genes, with 68.3% of these genes detected in the dominant STs and *bla*_OXA-181_ the most commonly co-carried carbapenemase gene (n = 110; 61.1%) (Figure 3A). Among the 446 ST410 isolates, 69 (15.5%) carried two carbapenemase genes, which was the highest proportion compared with other dominant STs (1.8–7.4%).

Single ESBL genes found in less than 20 isolates are categorised as “Other single ESBL genes”. Two or three ESBL genes are grouped as “2–3 ESBL genes”.

^a^ The overall dataset included isolates from the following countries: Argentina, Australia, Austria, Bangladesh, Cambodia, Canada, Chile, China, Czechia, Denmark, Egypt, Finland, France, Germany, Hungary, India, Ireland, Israel, Italy, Japan, Kenya, Laos, Lebanon, Malawi, Myanmar/Burma, Nepal, the Netherlands, Niger, Nigeria, Norway, Pakistan, Portugal, Qatar, Singapore, South Korea, Spain, Sweden, Switzerland, Taiwan, Tanzania, Thailand, United Kingdom, United States and Vietnam, collected or uploaded to NCBI between 2014 and 2022.

Many *bla*_NDM-5_-positive *E. coli* (n = 2,129; 62.0%) also harboured at least one extended-spectrum β-lactamase (ESBL) gene, with 62.3% of isolates being of the dominant STs (Figure 3B). The most frequently identified ESBL genes were *bla*_CTX-M_ family genes (n = 2,096; 98.4%) with most isolates positive for *bla*_CTX-M-15_ (n = 1,464; 68.8%). Plasmid-mediated AmpC β-lactamase determinants were also prevalent (n = 1,027; 29.9%), with 67.7% of them identified in the dominant STs. The most often identified genes encoding for acquired AmpC β-lactamases were* bla*_CMY-42_ (n = 490; 47.7%) and *bla*_CMY-2_ (n = 406; 39.5%). Of note, the highest proportion of *E. coli* isolates harbouring *bla*_CMY-2_ (n = 266; 65.5%) belonged to ST410 compared with other dominant STs (< 11% each).

High predicted resistance to aminoglycosides (n = 3,236; 94.2%) was observed among *E. coli* isolates carrying *bla*_NDM-5_, the most frequent genes being *aadA2* (74.9%), *aac(6’)-Ib-cr* (39.4%), *aadA5* (34.8%) and *rmtB* (24.6%). Predicted resistance to fluoroquinolones was also common based on one or more mutations in type II topoisomerase genes (n = 3,195; 93.0%). Combining the resistance markers, more than half (n = 2,000; 58.2%) of the *E. coli* isolates carrying *bla*_NDM-5_ in the overall dataset were predicted to be multidrug-resistant with resistance to all β-lactams (including aztreonam), aminoglycosides and fluoroquinolones for treatment of serious *E. coli* infections.

Similar trends were observed for predicted trimethoprim (n = 3,185; 92.7%) and sulfamethoxazole (n = 3,223; 93.8%) resistance, the most frequent genes being *dfrA12 *and* dfrA17* as well as *sul1* and *sul2*, respectively. Predicted resistance to colistin (plasmid-mediated *mcr-1.1* carriage: n = 176; 5.1%) and tigecycline (*tet*(X) carriage: n = 10; 0.3%) was low.

## Epidemiological findings

Among the 874 isolates from national collections, 618 (70.7%) had available information on infection/carriage status and 189 (30.6%) of those were associated with infection. Of 766 (87.6%) isolates with available information on sample type, 234 (30.5%) originated from clinical samples. The most frequent specimen type was urine (n = 178; 76.1%), while isolation from blood (n = 23; 9.8%) and the respiratory tract (n = 6; 2.6%) was infrequent. Of 341 *E. coli* isolates carrying *bla*_NDM-5_ with available information on prior travel and/or hospitalisation within the past 6 months before sampling, a link to a country outside of the EU/EEA was reported for 287 (84.2%) isolates, mostly countries in Asia (158/341; 46.3% isolates) or in Africa (125/341; 36.7% isolates).

## Discussion

Our results confirmed the emergence of *E. coli* isolates carrying *bla*_NDM-5_ in the EU/EEA initially detected in the CCRE survey and showed the predominance of five STs (ST167, ST410, ST405, ST361 and ST648) known as high-risk clones based on their rapid global spread and their association with virulence and multidrug resistance [8-16]. While these STs carrying *bla*_NDM-5_ have previously been described in the EU/EEA, reports were limited to a single ST in one [8,9] or two [10] institutions, or to the description of the national epidemiology of one single ST [11] or several STs [12,13]. Only one study including 33 isolates of *E. coli* carrying *bla*_NDM-5_ has previously reported cross-border emergence in Germany and Switzerland [14]. Our study included WGS data on 874 isolates from national reference laboratories in 13 countries and thus provides a more comprehensive analysis of the spread of these high-risk STs in Europe. Of the isolates with available information on prior travel and/or hospitalisation, 84.2% had a link with a country outside of the EU/EEA, mainly on the African and Asian continents, suggesting potential acquisition outside of the EU/EEA.

About 30% of the *E. coli* isolates carrying *bla*_NDM-5_ were documented as associated with infections, emphasising the clinical relevance and the need for early detection. Sixty-two per cent of isolates carried ESBL genes conferring resistance to aztreonam which is not inactivated by metallo-β-lactamases. Together with predicted resistance to aminoglycosides and fluoroquinolones, this leaves very limited options for the treatment of serious *E. coli* infections. With respect to aminoglycosides, the *rmtB*-encoded 16S rRNA methylase conferring resistance to novel aminoglycosides is a particular concern [12,17]. Moreover, studies have shown that isolates (over)-producing NDM-5 had increased minimum inhibitory concentrations to cefiderocol, one of the last treatment options for metallo-β-lactamase-producing Enterobacterales [18,19].

A limitation of this study is that it is not based on a standardised sampling protocol but on regular national surveillance with differences in sample collection protocols, national coverage and data completeness and that it is subject to detection and reporting bias. However, the frequent detection in 13 countries indicates that *E. coli* carrying *bla*_NDM-5_ is now established in the EU/EEA. The proportion of carbapenem resistance in *E. coli* in the EU/EEA has so far been very low, i.e. only 0.2% of invasive isolates [20]. Further spread of *E. coli* isolates carrying *bla*_NDM-5_ in community and healthcare settings, and within and between countries, is likely to increase dissemination with resulting higher levels of carbapenem and multidrug resistance in *E. coli* infections. 

## Conclusion

The spread of *E. coli* carrying *bla*_NDM-5_ is occurring rapidly and on a large geographical scale with a considerable risk for increasing carbapenem resistance in *E. coli* in the EU/EEA within a few years. Early detection and control are required to mitigate adverse consequences for patients and healthcare systems.
